# Self-Organising Maps and Correlation Analysis as a Tool to Explore Patterns in Excitation-Emission Matrix Data Sets and to Discriminate Dissolved Organic Matter Fluorescence Components

**DOI:** 10.1371/journal.pone.0099618

**Published:** 2014-06-06

**Authors:** Elisabet Ejarque-Gonzalez, Andrea Butturini

**Affiliations:** Departament d'Ecologia, Facultat de Biologia, Universitat de Barcelona, Barcelona, Catalunya, Spain; UMIT, Austria

## Abstract

Dissolved organic matter (DOM) is a complex mixture of organic compounds, ubiquitous in marine and freshwater systems. Fluorescence spectroscopy, by means of Excitation-Emission Matrices (EEM), has become an indispensable tool to study DOM sources, transport and fate in aquatic ecosystems. However the statistical treatment of large and heterogeneous EEM data sets still represents an important challenge for biogeochemists. Recently, Self-Organising Maps (SOM) has been proposed as a tool to explore patterns in large EEM data sets. SOM is a pattern recognition method which clusterizes and reduces the dimensionality of input EEMs without relying on any assumption about the data structure. In this paper, we show how SOM, coupled with a correlation analysis of the component planes, can be used both to explore patterns among samples, as well as to identify individual fluorescence components. We analysed a large and heterogeneous EEM data set, including samples from a river catchment collected under a range of hydrological conditions, along a 60-km downstream gradient, and under the influence of different degrees of anthropogenic impact. According to our results, chemical industry effluents appeared to have unique and distinctive spectral characteristics. On the other hand, river samples collected under flash flood conditions showed homogeneous EEM shapes. The correlation analysis of the component planes suggested the presence of four fluorescence components, consistent with DOM components previously described in the literature. A remarkable strength of this methodology was that outlier samples appeared naturally integrated in the analysis. We conclude that SOM coupled with a correlation analysis procedure is a promising tool for studying large and heterogeneous EEM data sets.

## Introduction

Excitation-Emission Matrices (EEMs) are three-dimensional fluorescence data that provide information about the composition of fluorescent chemical mixtures. They constitute optical landscapes that extend over the dimensions of excitation and emission wavelengths {λex–λem}, and where fluorophores appear in the form of peaks. In the field of marine and freshwater biogeochemistry, EEMs have been used for the study of dissolved organic matter (DOM), being a comprehensive analytical technique with which to characterise a highly complex mixture of organic compounds [1]–[3]. Indeed, EEMs have served to advance scientific knowledge about the ecology and biogeochemistry of DOM in aquatic systems [1], [2]. Most importantly, they have contributed to evidence that some fractions of DOM are highly reactive organic molecules that are involved in numerous ecosystem processes, such as bacterial uptake [4]–[6], metal binding [7], [8], photoreactivity [9]–[11] and light attenuation [12]. Overall these findings suggest the major involvement of DOM in the global carbon cycle [13], [14].

Despite the great potential for EEMs to increase knowledge about DOM behaviour in the environment, their interpretation and statistical treatment remain a challenge [15]. The spectral shapes of EEMs are complex mixtures of multiple and overlapping independent fluorescence phenomena, caused by the wide range of organic molecules contained in DOM. As only about 25% of these molecules have been identified [16], there is a lack of chemical standards to be used to separate the signal of bulk DOM into its individual components. For that reason, there is a need to develop pattern recognition methods capable of detecting and isolating the signal of different fluorescing moieties in the absence of any previous knowledge about the composition of DOM in a given sample.

A well-suited tool to satisfy these needs are Self-Organising Maps (SOM). SOM is an artificial neural network algorithm that mirrors the biological brain function [17]. Due to its unsupervised self-learning capacity, it is capable of recognizing patterns in complex data sets without following any assumptions about the data structure. Although it has been increasingly used within analytical chemistry in recent years [18] it has not been until recently that SOM has been used to analyse EEM data sets [19], [20], and the potential for SOM to equate or even outperform other state-of-the-art EEM data treatment methods like partial least-squares regression (PLS), principal components analysis (PCA) and parallel factor analysis (PARAFAC) has been highlighted [15], [18], [21], [22]. The map space produced by SOM offers multiple possibilities for the graphical representation of the output, allowing to unveil patterns among samples (best matching unit and unified distance matrices), as well as to explore what variables (wavelength coordinates in the case of EEM data sets) are the most influent in creating the sample patterns (component planes) [18]. However, pattern recognition at the variable level has remained at a qualitative stage, and the specific need to isolate independent fluorophores has not been covered.

Furthermore, previous analyses of EEM data sets with SOM were performed on data from engineered systems, where the diversity of fluorophores was essentially homogeneous among the samples [19], [20]. However, EEM data sets collected in natural water systems are subject to contain a wide diversity of spectral shapes, due to the multiple environmental factors that influence DOM quality [23]. In this case, data pattern interpretation may become more challenging, as the presence of outliers may alter the stability of the SOM output, and hence its reliability.

In this context, this study aims at expanding the evidences that SOM is a suitable tool for the study of EEM data sets. Specifically, we focus on two aspects. On the one hand, we aim to further test the performance of SOM when a high heterogeneity of spectral shapes is contained within the data set. We address this point by assessing the stability of the quantization and the neighbourhood relations of the SOM output under a leave-one-out cross-validation approach. On the other hand, we search for independent fluorophores by extending SOM with a correlation analysis of component planes. This constitutes a novel approach to discriminate areas of the EEM (i.e. groups of wavelength coordinates) representing different fluorophores.

## Materials and Methods

### Ethics statement

Some of the sampling sites included in this study were located in the protected areas of the Parc Natural del Montseny and Parc del Montnegre-Corredor, both under the authority of the Diputació de Barcelona. No specific permission was required to conduct the fieldwork. We confirm that our study did not involve any endangered or protected species.

### Data set

**Figure 1:**
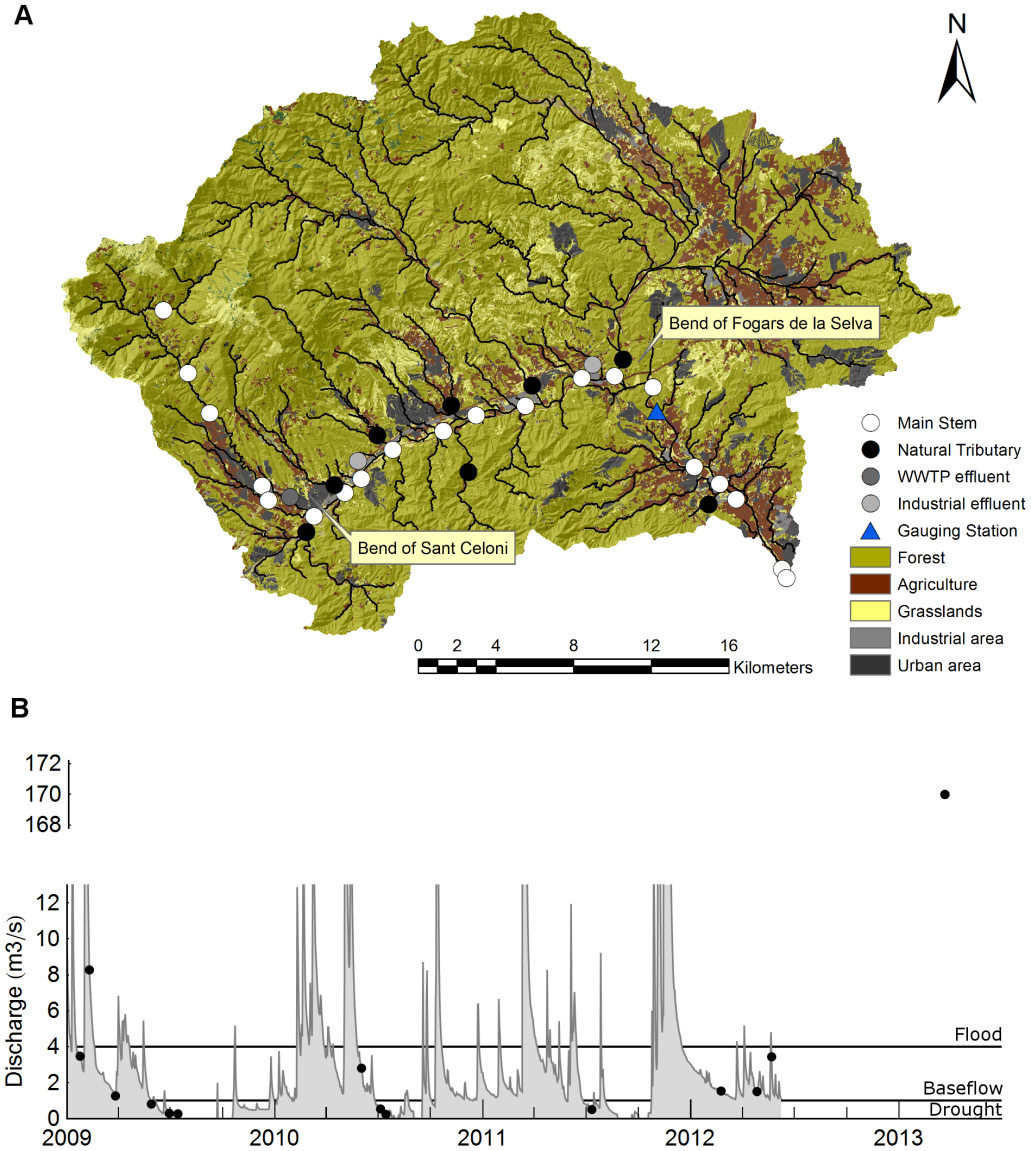
Experimental setting of the data set. A) Study site within the catchment from which the samples were collected. The river was operationally divided into three reaches: the “headwaters”, the “middle reaches” and the “lowland”. The divisions between segments correspond to the two big bends of Sant Celoni and Fogars de la Selva. B) Hydrogram contextualising the 15 sampling dates. Discharge data were recorded in the gauging station at Fogars de la Selva. Sampling dates were operationally divided into “flood” (Q>4 m^3^·s^−1^), “baseflow” (4>Q>1 m^3^·s^−1^) and “drought” (Q<1 m^3^·s^−1^) categories. As continuous monitoring was interrupted, the discharge on the last sampling date (2013/06/03) was measured individually on that date. All discharge data were provided by the Catalan Water Authority (Agència Catalana de l'Aigua, [24]).

Our EEM data set included 270 samples from a Mediterranean river catchment called La Tordera (865 km^2^), situated to the north-west of Barcelona, Catalunya. The sampling strategy was designed in order to assess the influence of space and hydrology on the EEM spectral shapes. Accordingly, in order to characterise the longitudinal dimension, water samples were collected at 20 sites along the main stem (60 km long). The sites were operationally categorised into three main reaches, referred to as “*headwaters*”, “*middle reaches*” and “*lowland*”, divided by the bends of Sant Celoni and Fogars de la Selva (Figure 1A). Each of these three river reaches has distinctive properties. The “*headwaters*” section corresponds to a forested catchment area with accentuated slopes and incipient human pressure, the “*middle reaches*” are characterised by intensive anthropogenic activity, receiving both diffuse inputs from urban activities and point source effluents of waste water treatment plants (WWTPs) and industries; and finally the “*lowland*” corresponds to a shallow and meandering geomorphology with a lower density of direct anthropogenic effluents. Eleven influent waters were also sampled upstream from the confluence with the main stem. Some of them correspond to natural tributaries with varying degrees of anthropogenic impact, whereas others correspond to WWTPs or effluents from chemical industries.

The seasonal hydrological variability was captured by sampling on 15 different dates during which a wide range of hydrological conditions was encountered: from flash floods to severe summer droughts (Figure 1B). In this case, samples were also operationally defined according to three categories: “*flood*” corresponds to discharges higher than 4 m^3^·s^−1^, “*drought*” to discharges lower than 1 m^3^·s^−1^, and “*baseflow*” to flows between 1 and 4 m^3^·s^−1^. We used discharge data from the gauging station of Fogars de la Selva, provided by the Agència Catalana de l'Aigua (Catalan Water Authority, [24]), as a reference.

Due to the wide variety of drained land cover, water sources and hydrological conditions included in the sampling design, the final EEM data set was expected to include a wide variety of spectral shapes.

### Field and laboratory procedures

Samples were collected in acid-rinsed glass bottles, and were kept refrigerated in the dark until arrival at the laboratory. Next, samples were filtered with 0.22-µm-pore nylon membranes and kept refrigerated until their spectral analysis, which was conducted within the next two days. Fluorescence analyses were performed using a Shimadzu RF-5301 PC spectrofluorometer equipped with a xenon lamp and a light-source compensation system (S/R mode). For every EEM, 21 synchronous scans were collected at 1-nm increments both in emission and in excitation. During each scan, excitation was measured over a wavelength range of 230 nm<λex<410 nm. Initial emission wavelengths ranged from 310 nm to 530 nm, at intervals of 10 nm. The bandwidth used for both excitation and emission was 5 nm. Spectra were acquired with a 1-cm quartz cell.

Absorption spectra were measured for fluorescence inner filter correction purposes using a Shimadzu UV-Visible UV1700 Pharma Spec spectrophotometer. Data were collected in double beam mode with wavelength scanned from 200 to 800 nm and with milliQ water as the blank. The slit width was set to 1 nm.

Raw EEM data were corrected and normalised to allow for inter study comparison following the steps described by Goletz et al. [25]. Spectral corrections were applied to both emission and excitation measurements to correct for wavelength-dependent inefficiencies of the detection system. An excitation correction function was determined using Rhodamine B as a quantum counter [26], whereas for emission a correction file was obtained by comparing the reference spectra of quinine sulphate and tryptophan provided by the National Institute of Standards and Technology (NIST) according to the procedure described by Gardecki and Maroncelli [27]. Next, data were normalised by the area under the Raman peak of a deionised water sample at λex = 350 nm and λem = {371–428} nm [28]. Inner filter effects were corrected by comparing absorbance measurements according to Lackowicz [26], as described by Larsson et al. [29]. Finally, a blank EEM of deionised water, measured on the same day of analysis and having undergone the same correction and normalisation procedures, was subtracted from every EEM sample.

### Optical indices calculation

Specific Ultra-Violet Absorbance (SUVA), as a surrogate measurement for DOC aromaticity, was measured as the Napierian absorption coefficient at λ_abs_ = 254 nm normalised by DOC concentration [30]. DOC concentration was determined by oxidative combustion and infrared analysis using a Shimadzu TOC Analyser TOC-V_CSH_.

The Humification Index (HIX), indicator of the humification degree of humic substances, was calculated as the ratio between the area under {λex_254_, λem_(435–480)_} and the area under {λex_254_, λem_(330–345)_}, as described by Zsolnay [31]. Finally, the Fluorescence Index (FI) [32], [33], indicator of the allochthonous vs autochthonous origin of DOM, was calculated as the fluorescence intensity at {λex, λem} = {370,470} nm divided by that at {λex, λem} = {370,520} nm.

### Self-organising maps

Self-Organising Maps (SOM) – also known as Kohonen maps – are a special type of two-layered artificial neural network (ANN). ANNs are mathematical models mirrored in the functioning of the biological nervous system, which have the ability to learn the patterns of input features and predict an output. They consist of an adaptive system of interconnected neurons – or processing units – that change their structure during a learning phase. In this phase, weight vectors (called prototype vectors or, in this context, prototype EEMs) that lie in the connections between neurons are adjusted to minimize the overall error of the network prediction [34].

By the end of the learning process, the EEM samples have been assigned to their best matching unit (BMU), that is, the unit that has the most similar prototype EEM. Thus, the outcome of the SOM will be a grid in which each unit will contain a prototype EEM whose spectral properties vary gradually but unevenly across the grid, according to the characteristics of the input data. By projecting the original EEMs on their BMU in the SOM grid, sample patterns can be explored.

According to Cattell [35], this analysis can be considered as an analysis in the Q mode, as it consists of a comparison between objects [36]. It can be seen as an exercise involving reduction of the dimensionality, in which samples become distributed over a two-dimensional grid, as well as a classification process, whereby samples become grouped into discrete units [37]. Moreover, in order to facilitate visual inspection of the distribution of the samples across the SOM grid, the analysis can be complemented with a clustering analysis of the neural EEM prototypes [38].

### Correlation analysis and the determination of EEM fluorescence components

In the SOM grid, it is possible to represent the intensity of a given wavelength coordinate of the prototype EEMs throughout the different neurons using a colour scale. This kind of visualisation is called a component plane [17], and shows how the fluorescence magnitude on a given coordinate varies from neuron to neuron over the SOM grid. Two highly correlated wavelength coordinates will therefore produce two similar component planes [39], [40]. When the number of variables in the data set is low, it is possible to visually compare the patterns among component planes and detect which ones are positively, negatively or not correlated [41], [42]. However, this becomes an unfeasible task when dealing with high-dimensional data, as is the case of EEMs (in our case, defined by 366 λex–λem coordinates). Barreto-Sanz and Perez-Uribe [39] proposed a methodology to simplify this task by projecting the correlations between the component planes on a new SOM grid. This new projection groups highly correlated variables into the same neuron, and moderately correlated variables into nearby neurons. At this point, a hierarchical clustering analysis can be used to determine a consistent number of groups of {λex–λem} coordinates, each of which can be considered as a different fluorescence component. As in this case the analysis involves exploring dependences between the descriptors, it can be considered as an R-mode SOM analysis [35], [36].

### Computations

**Figure 2:**
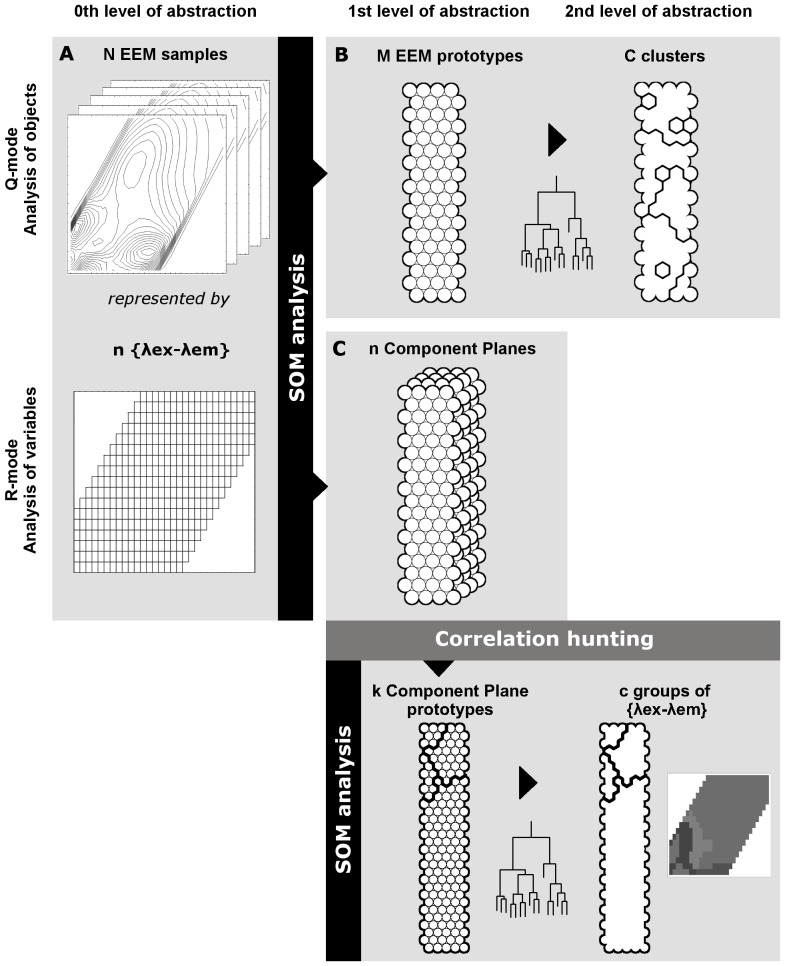
Summary of the methodology applied in this study. A) N initial samples are reduced to M prototype EEMs by SOM analysis. B) EEM prototypes are clustered to facilitate exploration of the relationships between the sample EEMs. C) SOM is performed on the correlation matrix of the component planes of the Q-mode SOM analysis. The output corresponds to an aggregation of highly correlated wavelength coordinates in a single neuron unit. D) Neuron units are clustered in order to find groups of highly correlated wavelength coordinates. E) Wavelength coordinate clusters are displayed in an EEM optical space in order to evaluate their biogeochemical meaning. Adapted and extended from Vesanto and Alhoniemi [38].

SOM analysis was conducted using the Kohonen package for R [37]. The successive steps undertaken in our computations are conceptualised in the flow diagram shown in Figure 2. EEMs were pre-processed by normalising their fluorescence intensity by their maximum, in order remove effects of changes in concentration and focus specifically on qualitative variations [43]. The input matrix for the SOM analysis in the Q-mode contained 270 linearized EEMs with fluorescence data from 366 λex–λem coordinate pairs (Figure 2A). The output layer was an hexagonal grid (Figure 2B). Its size was chosen to be the largest size that ensured stability of the quantization error [44]. In addition, dimensions were set to preserve the proportions of the two highest eigenvalues of the covariance matrix of the input data [19], [45]–[47]. During the training phase, the learning rate decreased linearly from 0.05 to 0.01. The initial neighbourhood size included two-thirds of all distances of the map units, and decreased linearly during the first third of the iterations. After that, only the winning unit was being adapted. In order to emphasise dissimilarities between the neurons of the SOM grid, a hierarchical cluster analysis with complete linkage was performed using the Lance-Williams update formula [48].

The influence of outliers on the performance of SOM was assessed by evaluating the quality of the SOM output in a series of leave-one-out (LOO) sample subsets. As measures of output quality, we used the SOM reliability criteria described by de Bodt et al. [44], which tested the stability of both the quantization and the topology of the SOM model. The stability of the quantization was assessed using the intra-class sum of squares (SSIntra) statistic, which is the sum of the squared distances between the observed data and their corresponding neural centroid. On the other hand, the stability of the neighbourhood relations was inspected by computing the histograms of all pairwise neighbourhood stabilities of a given LOO subset. SSIntra and neighbourhood stabilities were computed as described in de Bodt et al. [44]. For every LOO subset, the statistics were averaged over 50 runs of the SOM analysis, in order to minimise the variability of the output due to random initialisation of the reference vectors [49].

In parallel, 366 component planes were obtained from the SOM analysis (Figure 2C), one for each {λex–λem} coordinate that defined our original EEMs. In order to discriminate the number of fluorescence components within the samples, a correlation analysis was performed, based on the steps defined by Barreto-Sanz and Pérez-Uribe [39]. These steps included:

Transformation of the component planes into normalised vectors.
Calculation of the Pearson's correlation between each pair of vectors, obtaining a covariance matrix of dimensions (366×366).
Computation of a SOM analysis of this covariance matrix, hereafter referred to as the SOM analysis in the R-mode. In this grid, neurons grouped highly correlated {λem–λem} coordinates.
Clustering of the U-matrix with a hierarchical cluster analysis with complete linkage using the Lance-Williams update formula [48].
The optimal number of groups (i.e. fluorescence components) was determined by inspecting the silhouettes [50] of a range of partitions, from two to nine groups. The best partition had a high average 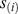, and the fewest objects with a negative 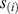, where 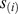 is a measurement of how well object 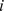 matches its assigned cluster.

Eventually, the correlation analysis led to the definition of a number of EEM regions containing uncorrelated fluorescence phenomena and hence, assumed to reflect different fluorescence components. Next, the components in every sample were quantified as area-normalised fluorescence volumes, following the Fluorescence Regional Integration described Chen et al. [51].

Finally, the fluorescence components found by correlation analysis, and expressed as normalised volumes as described above, were evaluated as descriptors of the data set by performing a non-metric multidimensional scaling (NMDS). The analysis was performed using the vegan package for R [52], and Bray-Curtis dissimilarities. Each variable was centred and scaled to a mean of 0 and a standard deviation of 1. In addition, the relationship between the fluorescence components and the optical indices of HIX, SUVA and FI was tested with a vector fit analysis within the NMDS ordination.

## Results

### SOM codebooks

**Figure 3:**
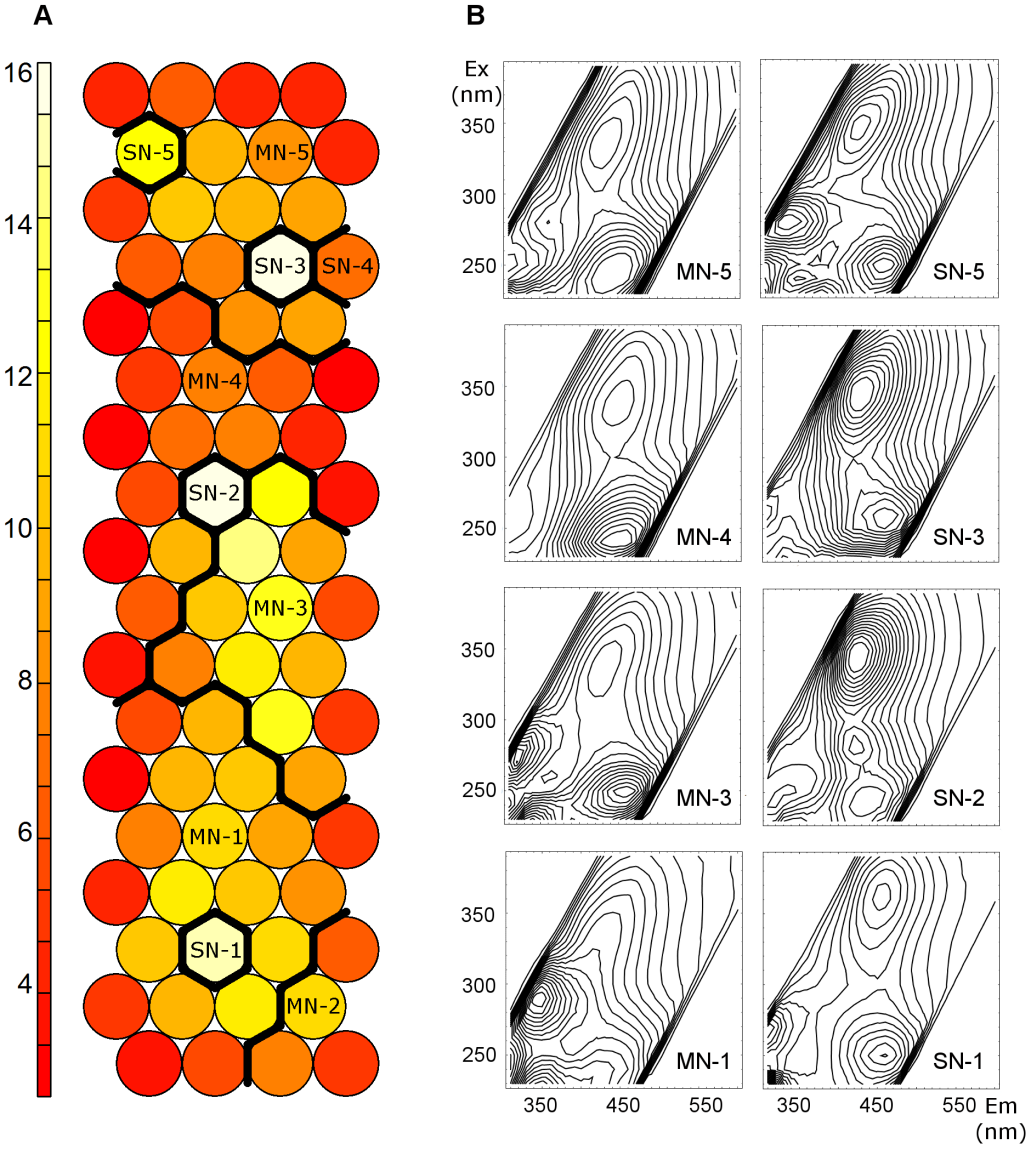
Clustering of the U-matrix of the SOM analysis in the Q-mode. A) Ten regions were defined in the SOM grid (black solid lines), based on hierarchical clustering of the U-matrix. B) EEM prototypes representing the main SOM regions.

The output of the SOM analysis trained on the 270-sample data set is summarised in Figure 3. The unified distance matrix (frequently referred to as U-matrix, Figure 3A) represents the distances between the EEM prototypes of neighbouring neurons using a colour scale [53]. This kind of visualisation is the most frequently used method to explore dissimilarity and clustering patterns in the SOM grid [17].

In our results, inter-neighbouring distances were clearly uneven across the SOM grid, indicating the presence of dissimilarity patterns. Low distances dominated in the upper-middle part of the U-matrix, whereas high dissimilarities were observed in the central region of the lower part of the SOM grid. In order to further emphasize and differentiate regions with higher similarities between neurons, a 10-cluster division was applied to the U-matrix (Figure 3A). It should be noted here that the partitioning of the U-matrix was used only for visualisation purposes. Some neurons had such a high dissimilarity to their neighbouring neurons (lowest values in the U-matrix) that they formed stand-alone clusters by themselves (hereafter referred to as SN-1 to SN-5, where SN stands for single neuron). The rest of the grid was partitioned into five multi-neuron zones (hereafter referred to as MN-1 to MN-5). The nomenclature specified in Figure 3 will be used hereafter to facilitate description of the distribution of samples throughout the SOM grid in order to explore relationships between samples.

### Outlier sensitivity analysis

**Figure 4:**
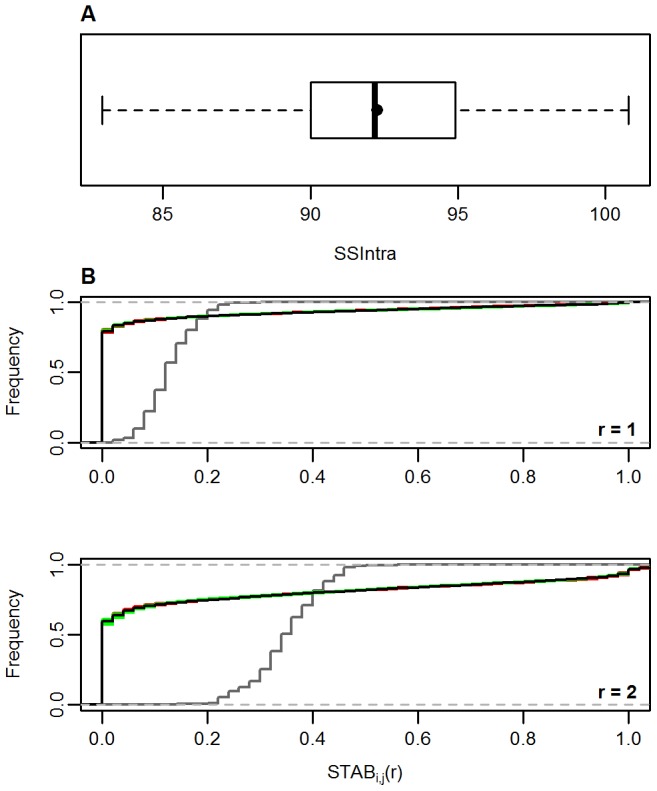
Outlier sensitivity test. A) Quantization stability: variation of the average SSIntra among 270 LOO subsets. The black dot indicates the mean. The absence of outlier values of CV(SSIntra) and the similar mean and median should be noted. B) Stability of neighbourhood relations: Histograms of the stabilities over all pairs of observations. In red, histograms of the LOO subsets in which the left-out sample was assigned to a single-neuron cluster. In green, histograms of the remaining LOO subsets. In black: histogram of the whole data set. It should be noted that there is hardly any difference between them. In grey, theoretical histogram of a randomly distributed map, following a binomial distribution defined according to de Bodt et al. [44]. This demonstrates that the SOM results are organised in a far from random distribution.

The outlier sensitivity test showed that the presence of a few samples with very distinctive and infrequent spectral shapes (especially those assigned to single-neuron clusters) did not affect the SOM outcome in a meaningful way. The SSIntra computed for the 270 LOO subsets followed a Gaussian distribution without any outlier values (Figure 4A). Moreover, the mean was almost identical to the median (92.27 and 92.17, respectively), further indicating that none of the LOO subsets exhibited a statistically relevant differentiated quantization structure.

The histograms of neighbourhood stability showed that at a radius of one and two neurons, the neighbourhood relations remained almost the same irrespective of the sample left out by the LOO subsets (Figure 4B). This demonstrates that the topology of the SOM output is preserved in the presence of specific outlier samples. Furthermore, all the histograms of the LOO subsets are clearly different from the theoretical histogram of a randomly organised map (Figure 4B). This indicates that in every SOM analysis, corresponding to different LOO subsets, the samples are meaningfully organised in the SOM grid, in a far from random distribution [44].

### Sample projection

**Figure 5:**
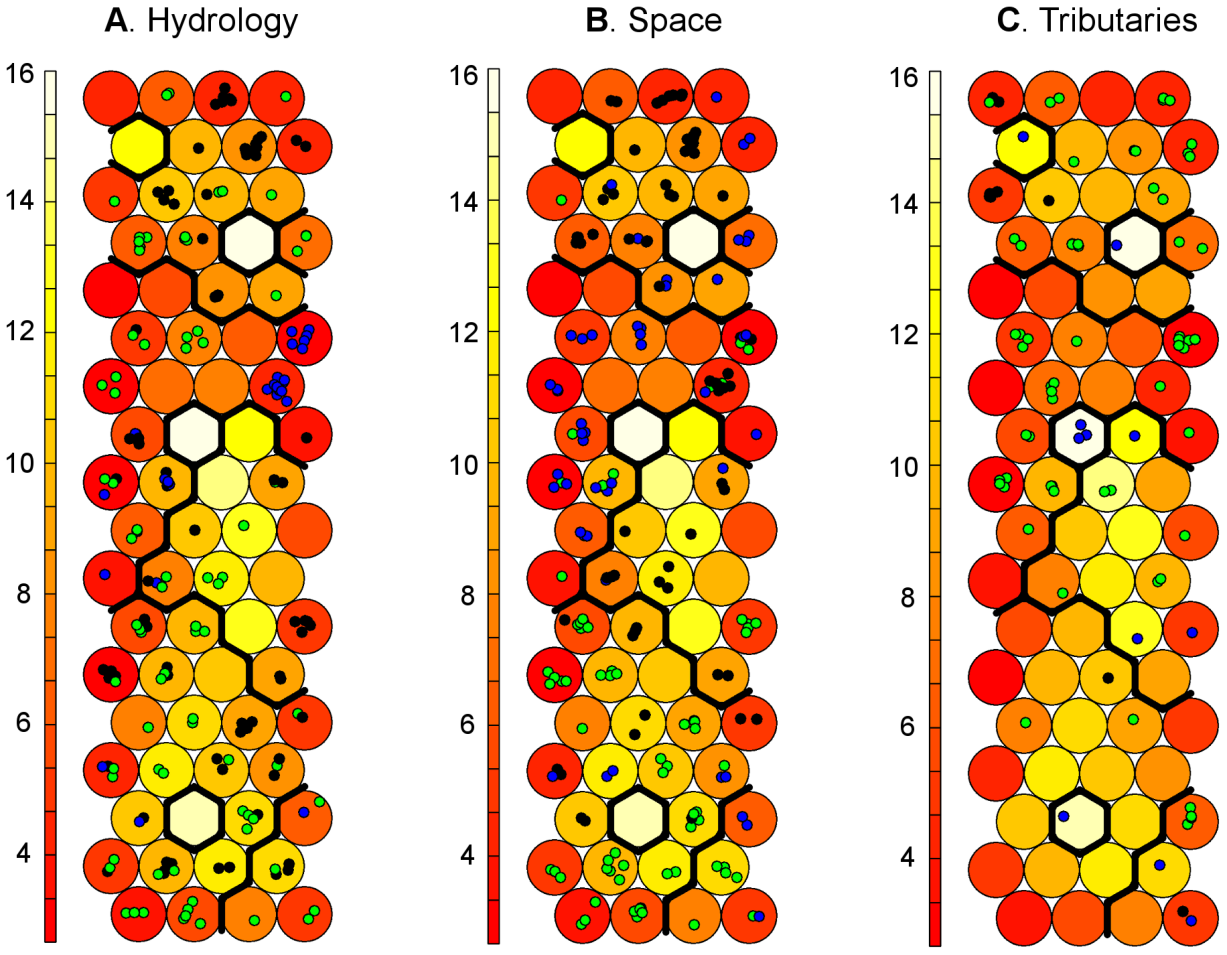
Projection of space, discharge, and type of tributary onto the U-matrix. Neuron colour scale indicates, for every neuron, the sum of the euclidean distances to all its immediate neighbours. Samples are projected on the SOM grid and coloured according to A) hydrology: blue represents flood conditions, black represents base flow, and green drought; B) space: blue corresponds to headwater samples, black middle reaches samples, and green are the lowland samples; C) types of tributary: blue are industrial, black are WWTP, and green are natural tributaries.

The samples in our data set were collected along a longitudinal downstream gradient, and under a variety of hydrological conditions. In order to test the influence of space and hydrology on the distributions of EEM spectral shapes, samples were projected onto the SOM grid, and coloured according to their sampling location (“headwaters”, “middle reaches” and “lowland” categories) and hydrology (“flood”, “baseflow” and “drought” categories, Figure 5).

In terms of hydrology (Figure 5A), samples collected during flood conditions were grouped into three main neurons, all situated in region MN-4. However, baseflow and drought samples were distributed across the grid. In the case of space (Figure 5B), the three categories appeared in different parts of the SOM grid. Headwater samples appeared mainly in region MN-4, samples from the middle reaches in regions MN-3 and MN-5, and those from the lowland mainly in region MN-1. Specifically, the neurons in region MN-4, which contained samples from middle reaches or the lowland, were the very same neurons that corresponded to the flood category in the hydrological projection. This combination of a single category for hydrology (flood) and multi category for space (whole length of the river) in a single neuron suggests a homogenisation effect on the spectral shape of EEMs over the whole length of the river under flood conditions.

Tributaries are presented separately in Figure 5C, coloured according to their origin: riverine, sewage-treated or industrial. It is noteworthy that single-neuron clusters contained exclusively industrial effluents, indicating that these sources produce DOM spectral shapes that are dissimilar with respect to the DOM from riverine and sewage-treated water. In contrast, WWTP samples appeared mainly in region MN-5, and natural tributaries were spread over the whole grid, but mainly in regions MN-4 and MN-5, those also associated with headwaters and middle reach sampling locations.

### Determination of fluorescence components

**Figure 6:**
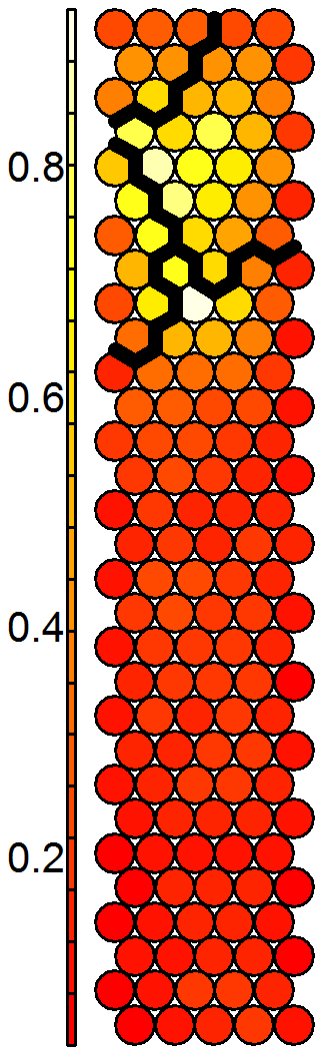
Clustering of the U-matrix of the SOM analysis in the R-mode. Every cluster groups highly correlated wavelength coordinates, representing different fluorescence components.

The U-matrix of the SOM analysis in the R mode is shown in Figure 6. It can be seen that the bottom half of the SOM grid contains highly correlated wavelength coordinates, expressed by the homogeneous dark red-coloured neurons that indicate short distances between them. In the top part, there is a central light-coloured region and darker neurons in the margins, indicating the presence of greater heterogeneity among these units. Hence, overall the SOM grid contains a high number of neurons with highly correlated wavelength coordinates, and in contrast, a small set of neurons with larger dissimilarities between them, thus containing a higher diversity of fluorescence signals.

Next, the hierarchical clustering and silhouette analysis of the SOM units showed that four clusters was the best number of fluorescence components, as it exhibited the optimal combination of the minimal number of presumably misplaced samples (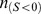) and the highest average silhouette (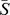), (Table 1).

**Table 1:** Characteristics of the silhouettes of a range of hierarchical partitionings of the R-mode SOM grid.

# groups	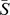	*S*_min_	*S*_max_	*n*_(*S*<0)_
2	0.56	−0.74	0.86	17
3	0.57	−0.50	0.80	13
4	0.54	−0.29	0.74	9
5	0.48	−0.43	0.72	13
6	0.48	−0.44	0.70	8
7	0.41	−0.23	0.70	7
8	0.42	−0.23	0.70	7
9	0.35	−0.33	0.70	16

The silhouettes analysis [5] corresponds to the calculation of 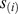 for every object in the data set, where 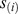 is a measurement of how well object 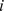 matches its assigned cluster. 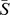 corresponds to the average 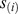, 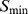 to the minimum 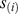, 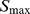 to the maximum 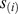 and 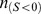 to the number of objects that have a negative 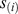. Values of S near one indicate that the object is very well clustered, whereas negative S indicates that the object might be assigned to the wrong group.

**Figure 7:**
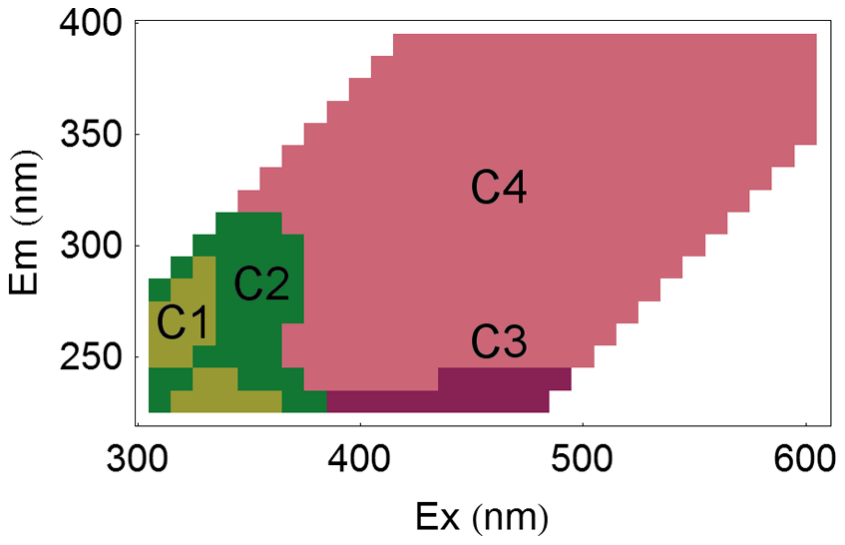
Localisation of the fluorescence components. Representation of the four groups of wavelength coordinates determined by correlation analysis on the excitation-emission space.

The four groups of wavelength coordinates (hereafter referred to as C1 to C4) are represented on the excitation-emission space in Figure 7. It can be seen that they appear spatially grouped in the optical plane and, moreover, that they overlap regions previously related to specific DOM fluorophores in the literature (Table 2). C4 corresponds to the V region of Chen et al. [51] and broadly to peak C of Coble [54], which were associated with humic-like substances. This component has been detected in a wide range of aquatic environments but mainly in waters draining forested catchments [2], and hence, represents an indicator of terrestrially derived DOM [54]. In the same emission range, but at the lowest excitation wavelengths, component C3 is apparent. Similarly to C4, it has also been associated with humic-like components of terrestrial origin but with a higher molecular weight and more freshly released character [2], [55]. In the region of the EEM with the lowest emissions are two spots centred at λex/λem = 230/330 nm and 270/310 nm (C1), similarly to the coordinates of maximal fluorescence of tyrosine [56]. Hence, components appearing at these wavelengths have been attributed to peptide material resembling or containing tyrosine, indicating the presence of autochthonous microbially derived DOM [57]. Finally, C2 covers an area surrounding the previous protein-like spots, overlapping the region occupied by tryptophan [56]. This component has also been reported to reflect microbial activity, and has been used as an indicator of anthropogenic DOM inputs [58]–[60].

**Table 2:** Wavelength coordinate boundaries of the fluorescence components.

Component	Correspondence with		Approximate boundaries	
	Coble 1996 [54]	Parlanti 2000 [62]	λex (nm)	λem (nm)
C1	B	γ	250–280 and 230–240	310–330 and 320–360
C2	T	δ	240–300	340–370
C3	A	α′	230–240	>370
C4	C	α	>250	>400

Summary of the location of the fluorescence components determined by correlation analysis and correspondence with previous components described in the literature.

### SOM fluorescence components as descriptors of the data set

**Figure 8:**
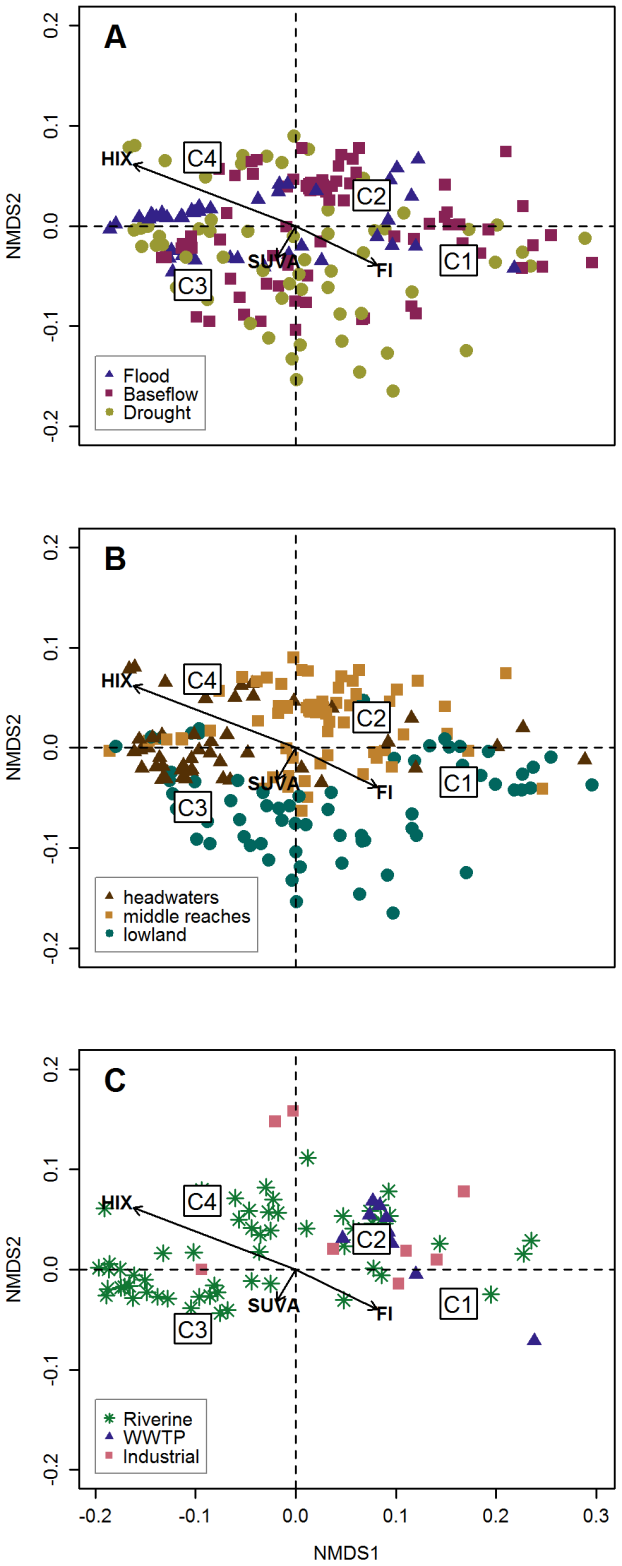
Multivariate analysis of our data set based on the four fluorescence components determined by SOM analysis. A non-metric multidimensional scaling was complemented with a vector fit analysis with the optical indices HIX, SUVA and FI. A) Main stem sites are coloured according to their discharge category. B) Main stem sites are coloured according to their downstream distance. C) Tributary sites are represented according to their source type.

Finally, we evaluated the capacity of these four fluorescence components to describe patterns in our data set as new independent variables by performing a NMDS. The results are shown in Figure 8. For the sake of simplicity in exploring the distribution of the samples in the NMDS space, panels A and B include only the main stem sites, whereas panel C includes only the tributary sites. However, it should be noted that all three figures come from the same analysis, and therefore the loadings of the variables (i.e. the fluorescence components C1 to C4) and the vector fit analysis of the optical indices is the same in the three panels.

In summary, the first axis separates the humic-like components C3 and C4 (negative side) from the protein-like components C1 and C2 (positive side). HIX and FI are oriented, respectively, in the negative and the positive directions of the first axis with a high level of significance (p<0.001). This reinforces our interpretation of the components, such that C1 and C2 are related to microbially derived components, whereas C3 and C4 are related to terrestrially derived components. The second axis separates C1 and C3 from C2 and C4, suggesting further differentiation within the protein- and the humic-like groups of components. SUVA appears directed towards C3, however with a weaker level of significance (p<0.05). It is noteworthy, though, that SUVA and HIX appear perpendicular, showing independency from one another, even though they have previously been found to characterise a similar aspect of DOM [61].

According to our sampling design, we checked the role of hydrology and space in this new ordination based on fluorescence composition. In panel A, objects are coloured according to the discharge category under which they were sampled. The samples collected during flood conditions appear clearly aligned between the region of C3 and C4 and that of component C2. Samples from baseflow and drought conditions appear more broadly distributed throughout the whole NMDS plane. Drought samples seem to be more dispersed and occupy the negative secondary axis, which is not directly associated with any fluorescence component or optical index.

In space, the most important segregation occurs on the second axis. The sites from the lowland appear on the negative side, whereas those from the headwaters and the middle reaches are found on the positive side. Furthermore, headwater samples appear slightly more concentrated in the region between C3 and C4, similarly to the situation for flood samples in panel A.

Finally, panel C shows the tributary sites, which comprise a mixture of natural and anthropogenic water types. This figure shows a very clear pattern, consisting of an aggregation of industrial and WWTP effluents near component C2. This suggests a relationship between C2 and anthropogenically derived DOM.

## Discussion

SOM coupled with a correlation analysis offers a flexible tool that enables, in the first stage, a similarity-based classification of EEMs and, in the second stage, a reduction of the dimensionality by grouping highly correlated {λex–λem} coordinates (Figure 2). Hence the methodology consists of two main parts: first, an analysis of the objects (i.e. sample EEMs) and second, an analysis of the variables (i.e. wavelength coordinates). In essence, the analysis of the objects is an exercise of classification of the samples, based on their spectral similarities; whereas the analysis of the variables reduces the dimensionality by grouping those coordinates that are highly correlated. This correlation analysis has meaningful biogeochemical implications, as each group of correlated wavelength pairs is assumed to be an independent fluorescent component, with consistent distributions in the λex–λem space according to the literature [54], [62].

As a classification system, SOM has the advantage that it shows a low degree of dependency on the frequency at which a sample (or a spectral shape) is represented in the data set. By means of an outlier sensitivity test, the SOM quantization and topological structure was found to be robust to the presence of outlier samples. Accordingly, a single sample with unique and distinctive features can be classified on its own without affecting the classification of the other samples. In this way, outliers are not a distorting element, but a result integrated into the whole output. In our data set, this was exemplified by the neurons SN-1, SN-3 and SN-5, each of which represented only one sample. Specifically, they represented industrial effluents, which had very different spectral shapes with respect to the river water samples. This robustness to outliers provides the advantage that a data set can be analysed irrespective of its heterogeneity. This circumvents the main limitation of other currently used and well-established methods for EEM data treatment, like PCA, PLS or PARAFAC, which are highly sensitive to the presence of outliers [63]–[65] as they largely depend on least-squares solutions [18]. In least squares methods, the overall model is adjusted to include a better fit of an outlier, even if it results in a lower overall fit [66]. However, in SOM every sample only modifies its BMU and its neighbourhood, resulting in a less apparent influence of the presence of an outlier on the whole model outcome.

Furthermore, this classification stage leads not only to the grouping of samples with a high degree of similarity in terms of spectral shapes, but also to the generation of a reduced number of EEM prototypes (Figure 2, 0th to 1st level of abstraction). This reduced data set contains all the initial diversity of spectral shapes, but with the relative frequencies more evenly distributed. For instance, in our work, one EEM prototype could represent either a large number of samples that were very similar to one another (e.g. 13 headwater samples in a single neuron in SOM region MN-4, Figure 5B), or just a single sample with very unique properties (e.g. an industrial effluent in SN-1, SN-3 or SN-5, Figure 5C). This re-weighting effect of the representativeness within the data set allows for an analysis of correlations among variables (i.e. λex–λem coordinates) that can detect fluorophores that were initially represented at only low levels. Indeed, in our correlation analysis, we distinguished four areas in the EEM that were highly correlated (Figure 2, 1st to 2nd level of abstraction). Our four components had consistent properties in relation to previous descriptions in the literature (Table 2). Specifically, we distinguished two protein-like components, one of which appeared specifically related to anthropogenically derived DOM, as well as two humic-like components that coincided with the A and C areas described by Coble [54].

This methodology for detecting fluorescence components represents a novel statistical approach. In the procedure, the partitioning of the SOM grid represents a key step where the final decision is taken about the number of fluorescence components present in the data set. This step requires particular attention. Specifically, there are several clustering techniques that could be used to classify the neurons in a SOM grid. It has been reported that SOMs create clusters similar to those created by hierarchical clustering [38], [67]. Indeed, we computed a hierarchical clustering with complete linkage using the Lance-Williams update formula, and our clusters were consistent with the (dis)similarity patterns of the U-matrix (Figures 3 and 6). However, in SOM grids of higher resolution (i.e., number of neurons) the U-matrix can present more complex patterns of clustering and subclustering. In this case, the results of a hierarchical clustering analysis may not follow the results of the U-matrix very closely [68]. As a better approximation, computation of Vellido's algorithm and the use of the U-matrix neural neighbourhood distances as a cluster distance function have been proposed [39], [68] as, in this case, the neighbourhood conditions become explicit in the analysis and the output fits better with the results of the U-matrix. Hence, future studies should test the performance of different clustering techniques when larger data sets – and hence, larger SOM grids – are concerned.

Finally, after the regionalisation of EEMs into four fluorescence components, we quantified their contribution in every sample using the FRI technique originally described by Chen et al. [51]. This technique has been widely applied to track changes in DOM composition [69]–[71]. It has the advantage that it integrates the whole shape of the EEM region and accounts for the fluorescence provided by shoulders and other spectral features that would be omitted if only the maximal value of the region was taken into account. However, it has recently been pointed out that the numerical method used for integration can have important consequences for the accuracy of the results. Specifically, the Riemann summation method proposed by Chen et al. [51] and used in this paper may result in the underestimation of the protein-like fractions, and in the overestimation of humic-like fractions [72]. In order to minimise this bias, future studies may consider the use of other methods, such as the composite trapezoidal rule or the composite Simpson's rule [72].

Despite the main focus being on the methodology, some biogeochemically meaningful information arose throughout the study. Hydrology and downstream distance were found to be relevant shapers of DOM spectral properties. Floods exhibited differentiated patterns with respect to baseflow and drought conditions. Floods appeared to have a homogenisation effect on EEM spectral characteristics, with a gradual shift downstream between the presence of humic-like components with high HIX and SUVA. This indicates the prevalence of terrestrial humic-like material along the whole length of the river that rapidly transfers to the coastal system with little chance of being transformed [73]. The presence of C2 with high FI indicates some impact of industrial and WWTP effluents during downstream transport [58], [74]. Outside flood conditions, samples collected from the headwaters, the middle reaches and the lowland could be distinguished from each other. They exhibited successively lower HIX and higher FI values from the headwaters to the lowland. This indicated a shift from terrestrial-like characteristics to an autochthonously generated DOM character during downstream transport. Furthermore, industrial effluents exhibited unique and distinctive properties with respect to the rest of the data set.

In summary, our results open a new viewpoint to the statistical treatment of EEMs. Thanks to its robustness to the presence of outliers, SOM can be applied to EEM data sets including both high- and low-represented spectral shapes. This may have important practical implications especially for the study of the biogeochemical behaviour of DOM in natural systems, as sampling designs will be less restricted to the requirements of the statistical treatment, and more adaptable to research needs.

## Conclusions

In this paper, the use of SOM in combination with a correlation analysis has been presented as a powerful method to deal with large and complex EEM data sets. Specifically, our findings indicate that:

SOM analysis coupled with a correlation analysis as described by Barreto-Sanz and Perez-Uribe [39] allows an analysis both at the object and at the variable level. Hence, it serves not only to explore the differences in fluorescence properties between samples, as shown by Bieroza et al. [19], [20], but also helps to identify particular fluorescence components, as shown herein.
It is robust to the presence of outlier samples. That is, samples with very distinct features are discerned while having little effect on the ordination and classification of the other samples. This distinct property makes it possible to work with heterogeneous data sets.
The correlation analysis performed on the SOM EEM prototypes has an enhanced capacity to detect fluorophores that are represented at only low levels in the original EEM data set.

Therefore, we conclude that SOM analysis coupled with a correlation analysis of the component planes expands the toolbox of the fluorescence DOM researchers by enabling the analysis of complex and heterogeneous EEM data sets. This may open new possibilities for advancing our understanding of DOM character and biogeochemical behaviour.
