# Correction: Self-Organising Maps and Correlation Analysis as a Tool to Explore Patterns in Excitation-Emission Matrix Data Sets and to Discriminate Dissolved Organic Matter Fluorescence Components

**DOI:** 10.1371/journal.pone.0131382

**Published:** 2015-06-22

**Authors:** Elisabet Ejarque-Gonzalez, Andrea Butturini

In Fig 7, the labels of the axes are interchanged. The authors have provided a corrected version here.

**Fig 7:**
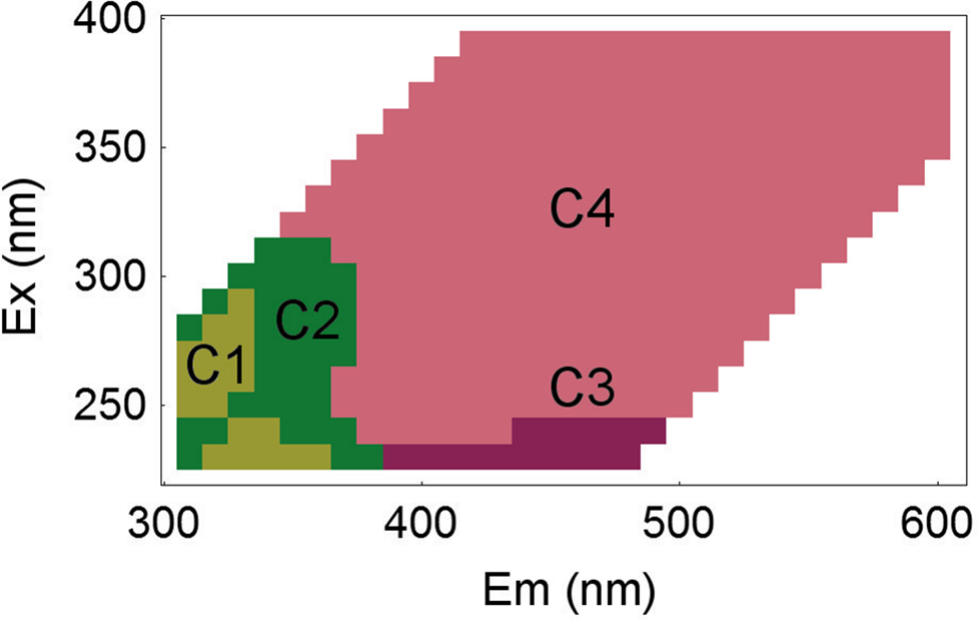
Localisation of the fluorescence components. Representation of the four groups of wavelength coordinates determined by correlation analysis on the excitation-emission space.
